# Process and Effects Evaluation of a Digital Mental Health Intervention Targeted at Improving Occupational Well-Being: Lessons From an Intervention Study With Failed Adoption

**DOI:** 10.2196/mental.4465

**Published:** 2016-05-11

**Authors:** Salla Muuraiskangas, Marja Harjumaa, Kirsikka Kaipainen, Miikka Ermes

**Affiliations:** ^1^ VTT Technical Research Centre of Finland Ltd Digital Health Oulu Finland; ^2^ VTT Technical Research Centre of Finland Ltd Digital Health Tampere Finland

**Keywords:** acceptance and commitment therapy, intervention studies, mHealth, cccupational health, process assessment, stress, mindfulness, attrition, adoption

## Abstract

**Background:**

Digital interventions have the potential to serve as cost-effective ways to manage occupational stress and well-being. However, little is known about the adoption of individual-level digital interventions at organizations.

**Objectives:**

The aim of this paper is to study the effects of an unguided digital mental health intervention in occupational well-being and the factors that influence the adoption of the intervention.

**Methods:**

The intervention was based on acceptance and commitment therapy (ACT) and its aim was to teach skills for stress management and mental well-being. It was delivered via a mobile and a Web-based app that were offered to employees of two information and communication technology (ICT) companies. The primary outcome measures were perceived stress and work engagement, measured by a 1-item stress questionnaire (Stress) and the Utrecht Work Engagement Scale (UWES-9). The intervention process was evaluated regarding the change mechanisms and intervention stages using mixed methods. The initial interviews were conducted face-to-face with human resource managers (n=2) of both companies in August 2013. The participants were recruited via information sessions and email invitations. The intervention period took place between November 2013 and March 2014. The participants were asked to complete online questionnaires at baseline, two months, and four months after the baseline measurement. The final phone interviews for the volunteer participants (n=17) and the human resource managers (n=2) were conducted in April to May 2014, five months after the baseline.

**Results:**

Of all the employees, only 27 (8.1%, 27/332) took the app into use, with a mean use of 4.8 (SD 4.7) different days. In the beginning, well-being was on good level in both companies and no significant changes in well-being were observed. The activities of the intervention process failed to integrate the intervention into everyday activities at the workplace. Those who took the app into use experienced many benefits such as relief in stressful situations. The app was perceived as a toolkit for personal well-being that gives concrete instructions on how mindfulness can be practiced. However, many barriers to participate in the intervention were identified at the individual level, such as lack of time, lack of perceived need, and lack of perceived benefits.

**Conclusions:**

The findings suggest that neither the setting nor the approach used in this study were successful in adopting new digital interventions at the target organizations. Barriers were faced at both the organizational as well as the individual level. At the organizational level, top management needs to be involved in the intervention planning for fitting into the organization policies, the existing technology infrastructure, and also targeting the organizational goals. At the individual level, concretizing the benefits of the preventive intervention and arranging time for app use at the workplace are likely to increase adoption.

## Introduction

Prevention of mental health problems is a topical issue. Psychosocial stress is a risk factor for mental health problems [1], and many serious medical conditions such as coronary heart disease [2,3]. Mental ill-health also causes an enormous economic burden and is becoming a key issue for the functioning of the Organization for Economic Cooperation and Development (OECD) labor markets and social policies [4].

Wider adoption of workplace wellness programs could prove beneficial for financial and productivity outcomes as well as health outcomes [5]. Occupational interventions usually aim to improve employees’ working conditions and/or health, reduce absences and employee turnover, and increase motivation and job satisfaction. Other objectives may include increased product quality, productivity, or customer satisfaction. Occupational interventions are evaluated by assessing the effects of planned activities at the worksites [6].

Work stress interventions utilizing acceptance and commitment therapy (ACT) have been shown to reduce stress and increase well-being and job performance [7,8]. ACT belongs to the third wave of cognitive behavioral therapies and emphasizes mindfulness and acceptance skills. The core concept of ACT is psychological flexibility, which refers to the ability to focus on the present moment and take actions that are aligned with personal goals and values even in uncomfortable or distressing situations [9-11].

Recently, digital interventions have been presented as more cost-effective and scalable means to promote health and well-being compared to face-to-face interventions [12]. Employers have started to incorporate Web-based approaches into their wellness programs, because the Internet provides an efficient avenue to reach and engage a large number of people [13]. Mobile apps could be especially suitable in well-being management in everyday life because they are easily accessible [14]. Digital interventions also have the potential to influence health and well-being at workplaces. However, the evidence of their effects on stress is still scarce. Until now, studies of preventive digital interventions have largely focused on physical health [15]. Although physical activity interventions can have a positive impact on both physical and mental health [16], apps focused on preventing mental health problems could be more effective in terms of stress management. Luckily, the importance of mental and social well-being has been acknowledged recently, and various systems have been developed specifically for the treatment of mental disorders [17].

Adoption processes of digital interventions at organizations have received little attention. In the context of this paper, the term adoption refers to a process that ends in the appropriate and effective use of a technology or digital service. In other studies the term operationalization has been used instead, referring to the actual introduction, adoption, and employment of the technology in practice, including also training and education [18]. Most Web-based or mobile interventions are primarily self-guided programs that are used by individuals who seek health or mental health-related support [19]. When such programs are introduced into a workplace, their adoption is influenced by organizational goals and stakeholders on higher organizational levels [20]. Due to mixed success of interventions in organizations, process evaluations explaining why the intervention succeeded or failed are advocated [21,22]. They can help to understand the entire process for creating successful organizational interventions. Even if an intervention program has been shown to be efficacious in controlled trials, the benefits will not be realized in the real world context unless implementation, adoption, and maintenance in an organization are successful.

Adoption of digital interventions should not be considered only from the organization’s point of view, but also from the individual’s point of view. Employees’ adherence, including the extent to which individuals experience the content of the intervention [23], has to be high enough to create successful outcomes. Users’ motivations and experiences play a central role in adoption because they affect people’s mental models and behaviors, and therefore the intervention outcome. The more people are intrinsically motivated, the higher the probability is to engage a person in long-term changes in behavior [24]. Documenting the experiences of the participants receiving the intervention helps to explain how and why changes were or were not achieved [25].

The aim of this paper is to evaluate the adoption process and effects of an individual-level unguided digital mental health intervention at organizations. In addition, it explores users’ motivations and experiences related to the mobile and Web app used to deliver the intervention. An earlier pilot study showed that the app can be acceptable, useful, and engaging among stressed working-age adults [14]. Thus, the two hypotheses of this study are (1) employees take the technology into active use supported by the organization; and (2) the intervention has a positive impact on employees’ well-being.

## Methods

### Intervention Evaluation

**Figure 1:**
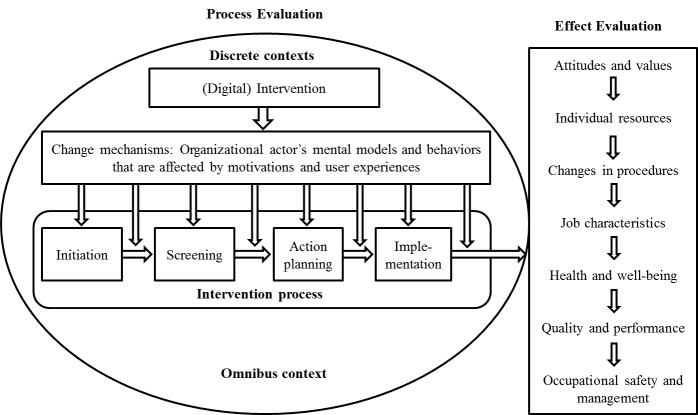
Intervention evaluation framework [22].

Nielsen and Abildgaard’s intervention evaluation framework was used for intervention process and effects evaluation [22]. The framework, where we also highlight the central components including digital intervention, motivations and user experiences as part of the change mechanisms are illustrated in Figure 1. The results are structured first with process evaluation including change mechanisms and intervention process. Then the effects evaluation is done on the effects on well-being.

### Intervention Context

#### Organizations

The intervention took place in two companies (Company A and Company B) working in the information and communication technology (ICT) industry (Table 1). At the time of the planning of the intervention, Company A was growing and had a higher employee turnover than Company B, which had reduced the number of personnel during the recent years. The companies did not have any previous experience on adoption of individual-level digital interventions. The purpose of the study was to evaluate the intervention process and effects within a company, not between companies.

**Table 1:** Description of the companies.

	Company A	Company B
Number of employees	230 in Finland	102 in Finland, over 4000 worldwide
Offices	2 locations	2 locations
Age of employees	35 years on average	30-40 years on average
Gender	83% men	28% men
Most common job titles	Software designer, consultant, project manager	Technical writer, consultant, project manager
Amount of remote work	Occasional remote work and travel	Technical writers often work at customers’ premises and consultants travel often
Amount of sick leaves	3-4% of working days, length of a sick leave usually 1-3 days	5-6% of working days, also some long sick leaves
Top 3 reasons for sick leaves	Common cold, stomach flu, musculoskeletal problems	Common cold, musculoskeletal problems, mental problems
Occupational health care	All employees are within the occupational health care	All employees are within the occupational health care
Work well-being assessments	Annual survey	Annual survey

#### Digital Intervention

**Figure 2:**
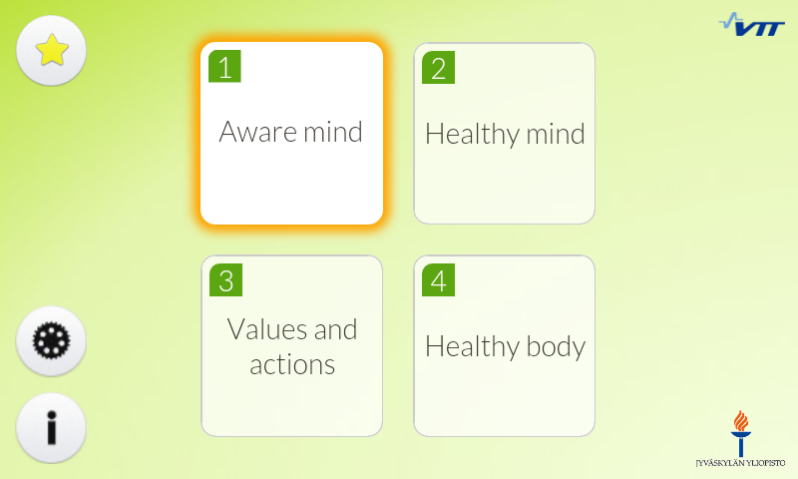
Screenshot of the mobile app (texts translated from Finnish).

**Figure 3:**
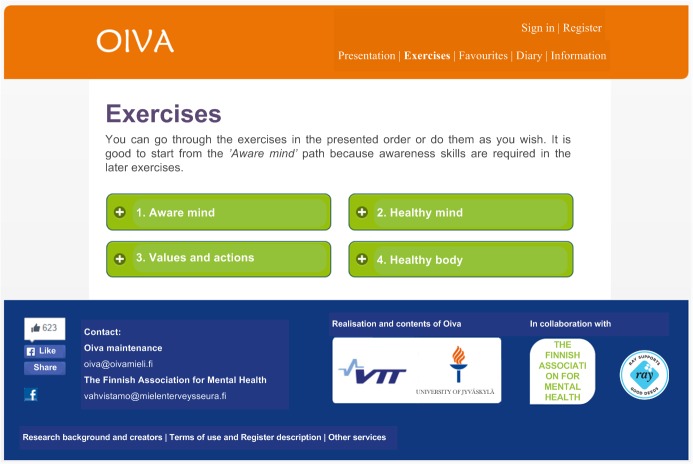
Screenshot of the web app (texts translated from Finnish).

The intervention content was delivered through a digital training app which was available in a mobile (iOS and Android) and a Web-based version (Figures 2 and 3) [14]. Participants received a link [26] for the app in the info sessions, emails, company intranet and flyers. Both apps were available only in Finnish. The app was based on ACT and targeted at stress management and mental well-being, teaching ACT skills in bite-sized sessions that could be listened to or read. The app was originally designed in cooperation between experts in user-centered design, psychology and computer science [14]. Its effects on stress have been studied in a randomized controlled trial [27], the results of which are not yet published.

#### Participant Recruitment

The participants of the study were the human resource (HR) managers and employees of the two ICT companies. The employees of both companies with sufficient computer literacy and fluency in Finnish were eligible to participate in the study. The HR managers of the companies invited the employees via email messages to an initial info session, which was held by researchers. The session consisted of a 20-minute presentation about the ACT approach, the app, and the aims and procedures of the study. In Company A, two info sessions were held at the two company offices. In Company B, the info session was arranged as an online meeting. In addition, the HR managers put information about the study in the company intranet and distributed paper flyers at the company offices. In all invitations and recruitment materials, the study was framed as a positive opportunity to improve one’s well-being and learn new skills to manage stress. The participants were instructed to use the digital app regularly, in brief sessions several times per week, but the intervention was otherwise unguided.

### Intervention Design

The intervention was designed to follow the stages outlined in Table 2 according to Nielsen and Abildgaard [22]. The delivery of the intervention was planned to be similar in both companies, with only small adaptations resulting from the information gathered during the initiation and screening stages.

**Table 2:** Intervention process design based on Nielsen and Abildgaard’s framework [22].

Stage	Tasks	Program design
Initiation	Clarify roles of different actors; create a communication strategy	First contact with companies through their HR managers
Screening	Assess organizations’ needs; select measures on individual and organizational level	Interviews with HR managers at the companies to recognize organization-specific needs and context; selection of suitable measures that complement existing assessment methods in the companies, with emphasis on measures focused on strengths and skills rather than problems and weaknesses
Action plans	Clarify intervention activities, their purpose and timeline; select methods for evaluating success of actions	App available on Web and mobile platforms; baseline survey conducted online; approximate intervention duration 3 months; kick-off events for employees held by researchers on-site and/or through online meetings; email and calendar invitations, intranet announcements and flyers at worksite; if possible, events aligned with other events/trainings in the company
Implementation	Document intervention activities; assign person who makes intervention happen in the organization	Regular contact with HR managers to keep track of the progress and activities inside the companies; mid-survey (online) to assess initial engagement and experiences among employees
Evaluation of effects	Measure changes in health and well-being; measure changes in working conditions and organizational procedures	Follow-up survey (online); analysis of changes in different well-being measures from baseline to follow-up; interviews with volunteer employees, superiors and HR managers

### Ethical Considerations

The study and all the related questionnaires were reviewed by the Ethics Committee of the Pirkanmaa Hospital District. Participants’ informed consent was obtained in the beginning of the baseline questionnaire online. The data was stored on a secure server. All the data was anonymized for reporting and publication.

### Data Collection

Data was collected between August 2013 and May 2014. The study began in August 2013 with HR managers’ interviews, and the intervention took place between November 2013 and March 2014. The study ended with the final interviews with the participants in April to May 2014.

Electronic questionnaires were sent via LimeSurvey (version 2.00) in the beginning of the study for all employees, and at two and four months for those employees who completed the baseline questionnaire. Occupational health and well-being was assessed in the beginning and in the end using a 1-item Stress questionnaire (Stress) [28], and a 9-item Utrecht Work Engagement Scale (UWES-9) [29,30] as primary outcome measures. The scores in the Stress questionnaire range from 1-5 with higher scores signifying a worse stress situation, whereas scores range from 0-6 for the UWES-9 test, where a higher score signifies a better work engagement. The Stress scale has shown satisfactory content, criterion, and construct validity for group level analysis [28]. The internal consistency of UWES-9 is good, with Cronbach's alpha varying between .85 and .92 across samples in 10 different countries [31,32].

Secondary outcome measures were an 84-item work-related well-being questionnaire which assessed both personal and work community well-being (P-TyHy, TyHy) [33], a 5-item Satisfaction With Life Scale (SWLS) [34], a 14-item Mindfulness questionnaire (FMI) [35], and a 7-item Work-Related Acceptance and Action Questionnaire (WAAQ) [10]. Motivations related to participation in the study and using the app were studied in the beginning, in the middle of the study, and in the end using ad hoc 16- and 14-item Self-Regulation Questionnaire (SRQ) [36]. Because an existing SRQ questionnaire for this specific context did not exist, questions were adaptations of the existing SRQ questionnaires. Motivations were inquired with additional open-ended questions. Details of the questionnaires and scales are shown in Table 3.

**Table 3:** Details of the secondary outcome measure questionnaires and scales.

Name	Score range	Significance of higher scores	Internal consistency	Cronbach's alpha range
Work-related well-being questionnaire (P-TyHy, TyHy)	0-70	Better occupational health and well-being	N/A	N/A
Satisfaction With Life Scale (SWLS)	5-35	Higher satisfaction with life	Good	.79-.89
Mindfulness questionnaire (FMI)	14-56	Higher mindfulness skills	Good	.86-.93
Work-Related Acceptance and Action Questionnaire (WAAQ)	0-42	Higher psychological flexibility	Good	.81-.84
Self-Regulation Questionnaires (SRQ)	1-7	Higher motivation	N/A	N/A

Qualitative data consisted of several semi-structured interviews. Before the intervention, HR managers (n=2) were interviewed separately, face-to-face for the companies’ characteristics, needs, and well-being challenges. In addition, the schedule for the intervention activities was outlined together with them. After the intervention, the HR managers were interviewed for their views on the success of the intervention, attitudes toward occupational health and mobile coaching in an organizational context, as well as intervention adoption in organizations. It should be noted that the HR manager of Company A changed during the course of the study.

After the intervention, a voluntary subset of employees (n=17) were interviewed, including 13 employees who had participated in the intervention study and 4 who had not participated. The interviewees were enquired about their experiences with the intervention process, motivations to learn stress management skills and participate in the study, attitudes toward occupational well-being, and mobile coaching in an organizational context. The intervention participants were also enquired about the motivations and user-experiences related to the app use. If an interviewee was working as a manager, such as team manager, additional questions were presented concerning the possibility of app use in team meetings and benefits of the app for managers. Interviews were conducted as 30 to 60 minute recorded phone interviews, after which notes were written and significant parts transcribed.

Data about the app usage was collected via app logs. When signing the informed consent, participants received a study identification code which was used to link the intervention usage logs to the participant. The participants were asked to input their code into the app settings so that their usage data could be identified. The usage log of the app, including time stamped user actions, was transmitted to a database on a secure server.

### Data Analysis

#### Quantitative Data Analysis

Baseline and final questionnaires were considered in the data analysis. The scales were analyzed, and their median values and inter-quartile ranges calculated. Statistical tests were conducted with IBM SPSS Statistics (version 20) software. Change in participants’ ratings of well-being was analyzed with a non-parametric Wilcox test and differences between the companies were analyzed with a non-parametric Mann-Whitney test. Well-being results were analyzed separately for both companies, and all together to evaluate the overall intervention. For the motivation items, the 7-point Likert scale was additionally dichotomized into agree or disagree for further analysis; persons answering 1-3 were considered disagreeing and 4-7 agreeing.

#### Qualitative Data Analysis

For the qualitative data, the overall evaluation was made by combining both populations due to the small sample size. The questionnaires’ open-ended questions and data from the final interviews were processed by putting the information from written documents into tables (MS Excel 2010, version 14) according to the original themes of the questionnaires and interviews. After this phase, common themes related to adoption as well as participants’ motivation and experiences were identified. In identifying motivations, themes arising from Self-Determination Theory (SDT) were used [24]. New categories and themes were created based on the data using principles of inductive content analysis.

#### Log Data Analysis

Usage logs were processed to calculate the usage duration in days and unique uses of the app. The duration was calculated as the time between the first and last timestamps. Actual use days, meaning the number of days when the app was used, were calculated.

## Results

### Participant Flow and Intervention Adoption

**Figure 4:**
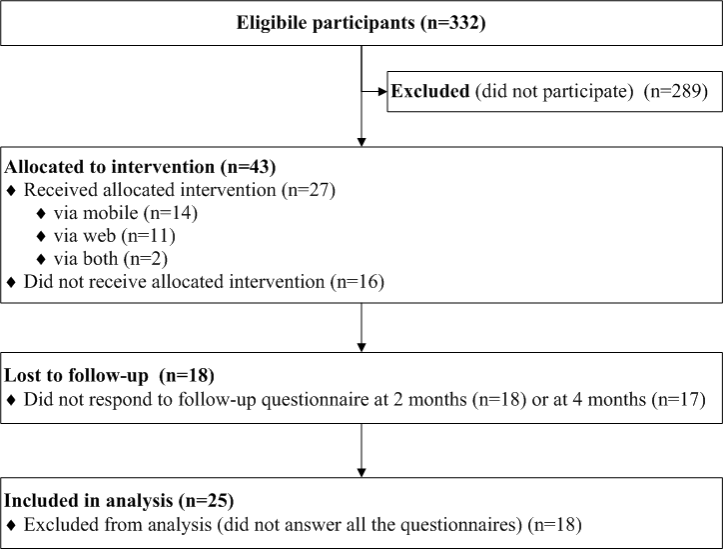
Participant flow diagram.

In total, 13.0% (43/332) of the employees participated in the study (Figure 4). The mean age of the participants was 37 years (range 25-54) and 67% (29/43) were female. It was found that 63% (27/43) of the participants, that is 8.1% (27/332) of all the employees started using the app, and 43 (13.0%, 43/332) answered the baseline questionnaire, 25 (58%, 25/43) the mid-term questionnaire, and 26 (60%, 26/43) the final questionnaire. Of those, 25 (58%, 25/43) participants answered all the questionnaires and were included to the longitudinal statistical analyses.

From the identified 25 logs, the mean use of the app was used 4.8 (SD 4.7) different days (range 0-19) and the mean app use period was 65.7 (SD 60.3) days (range 0-154). The app use period means the time between the first use session and last use session. For 64% (16/25), the use period was over week.

### Process Evaluation

#### Intervention Process

Since the face-to-face interviews in the beginning of the study provided largely similar information from both companies with respect to the companies’ characteristics, needs, and well-being, the intervention was implemented in a similar manner in both companies. However, there were minor deviations in the informing of the intervention. In Company B, the employees were more thoroughly informed of the upcoming study by the monthly letters and the participation of a researcher in one of the company’s remote meetings. Furthermore, the company culture, motivational factors, and challenges were somewhat different, and therefore, the information sessions emphasized different benefits, namely personal well-being and new skills in Company A and occupational well-being and helping others in Company B.

The findings show the intervention process applied in this study did not lead to successful adoption of a digital intervention in occupational well-being: only 13.0% (43/332) filled in the baseline questionnaire and only 8.1% (27/332) of all employees started using the app. In the following sections, the assumptions which the intervention design was based on, the correct assumptions (italics), and a clarification of where the assumptions failed are described.

##### Initiation

The assumptions in the beginning were that the HR managers (1) *are responsible for employees’ training, development, and actions that influence their overall performance and occupational health and well-being*; and (2) are authored to make relevant decisions or actions. The second assumption was incorrect; the top management made the final decisions about participating in this study and that employees could participate in kick-off meetings during working hours, but the app use had to take place on their own time. Although the HR managers were a rich source of information and a suitable first point of contact in the companies, more effort should have been taken to involve the top management because they made the strategic decisions.

##### Screening

The assumptions in the beginning were that (1) *the HR managers know the well-being situation in the company best;* (2) *they can provide contacts to other organizational actors as well as occupational health care;* (3) *they can benefit from the intervention personally;* (4) combining study measures with assessment tools already in place (current context) makes participation easier for the employees; and (5) positive tone in the questionnaires attracts more participants and can work as an intervention already by itself. The second assumption was only partially correct; contact between the occupational health care and the researchers was never established. The third assumption was correct but it was found that the app could also be extended to provide support for the HR managers in their work, such as leading for health and well-being. The fourth assumption could not be tested because it was not possible to combine the study measures with the existing ones in the organization due to conflicting schedules. The fifth assumption was incorrect; questionnaires with a positive tone and an instant feedback after submitting the questionnaire did not encourage people to stay in the study which can be seen in the high dropout rate (approximately 40%).

##### Action Plan

The assumptions in the beginning were that (1) aligning the intervention activities with existing activities in the company makes the process simpler for the employee; and (2) *multiple channels for recruitment and intervention content delivery reach more people than a single one.* The first assumption was not possible to be followed in the intervention realization because no other relevant ongoing activities could be identified within the proposed timeline of the study. The second assumption was correct but it has to be noted that not all channels were equally efficient. The paper brochures did not reach many employees, and a message from the HR and the management was mentioned to have more impact on participation.

##### Implementation

The assumptions in the beginning were that (1) the HR managers pay attention to intervention penetration; and (2) *the midterm questionnaire also works as a reminder for participants.* The first assumption was incorrect; since the intervention was not visible in the everyday life at the workplace, the HR managers did not know who took the app in use and did the exercises. Moreover, the HR manager of Company A changed during the study, which meant that the HR manager who was interviewed in the end had not been involved from the beginning of the study. The second assumption was correct, but even though the questionnaires reminded about the use of the app, the effects unfortunately did not realise in higher use activity.

##### Evaluation of Effects

The assumptions in the beginning were that (1) three months is a sufficient time to detect meaningful changes in well-being indicators; and (2) introduction of the intervention leads to changes in organizational procedures. The first assumption was not possible to test with such a small study population and low adherence. The second assumption was also incorrect. The intervention had no observable impact on organizational procedures.

#### Change Mechanisms

This section describes findings regarding individuals’ motivations related to study participation, digital intervention use, and user experience. The findings suggest that autonomous regulations were strongly present in both motivations to participate in the study and in the motivations to use the digital intervention. This is important since it is the most beneficial type of motivations in terms of long-term behavior change.

##### Motivations and Barriers to Participate in the Intervention

Motivations to participate in the intervention were inquired in the beginning of the study with an ad hoc SRQ. The most common reasons to participate in the study among the respondents (n=42) were (1) desire to feel better at work (100%, 42/42); (2) desire to improve well-being (100%, 42/42), (3) desire to learn new skills (98%, 41/42); (4) enjoyment of trying something new (93%, 39/42), (5) desire to advance scientific research (86%, 36/42), (6) interest in participating in a research study (86%, 36/42), (7) interest in figuring out how the digital intervention works (76%, 32/42); and (7) feeling good about doing something for the good of the society (67%, 28/42). Interviews highlighted similar motivations.

Barriers for participating were inquired with a separate mini-questionnaire from the non-participants (n=62) and they reported not participating in the study mainly because they (1) did not see problems in their occupation well-being and thus, they had no need for the app (68%, 42/62); (2) did not have time (65%, 40/62); and (3) did not remember to participate, which implies that the study was not in high priority for them (48%, 30/62).

##### Motivations and Barriers to Use the App

Motivations to use the app were inquired in the midterm questionnaire during the study with an ad hoc SRQ. The most common reasons to use the app among the respondents (n=18) were (1) importance of the personal well-being (100%, 18/18); (2) interest in making life changes (100%, 18/18); (3) desire to learn new things (100%, 18/18); (4) appreciation towards the app contents (94%, 17/18); (5) it was fun to do exercises with the app (83%, 15/18); and (6) it brought enjoyment to process everyday issues with the app (72%, 13/18).

Barriers for use were inquired in the midterm questionnaire from the participants who did not use the app. The most common reasons among the respondents (n=7) not to use were (1) not finding the time (100%, 7/7); and (2) not having a suitable phone (71%, 5/7) (eg, Windows phones were popular in Company B). Interviews confirmed that lack of time, having no need, and not remembering (ie, low priority) hindered app use. As one project manager from company B commented: "As a mother of small children, my time is limited."

Participants suggested several ways to support app use, such as group meetings, common use sessions at a workplace, reminders, follow-ups and provision of information on study progress.

##### User Experience Findings

User experiences were collected in the end of the study from the 13 interviewees who had used the app relatively actively.

The participants experienced that using the app helped mindfulness to become part of their routines, because the exercises gave concrete instructions how mindfulness can be practiced. They learned ways to perform breathing and relaxation exercises and the app made them aware of the importance of being present. As one team manager from Company B commented: "From that you remember those things and there are good phrases which are good to remember that ‘oh yeah, this is exactly how it goes’, kind of the awareness and reminding that these are good things."

However, participants, who were already familiar with mindfulness, experienced that the app did not bring anything new to them.

The app was seen as a toolkit for personal well-being. It helped in stress management, removed anxiety, brought relief in stressful situations, and helped to concentrate (eg, on themselves and their well-being) through improved mindfulness skills. As one project manager from Company A commented:

I got positive feelings (about this). It gave such an impression that if I would like to think about a small relaxation in the middle of the day, it would help and it could provide tools for this.

However, participants often stated they had used the app so little that it did have a noticeable influence on their well-being yet, despite feeling that intervention had a positive impact on the underlying factors of stress.

The app was mainly found to be easy to use. As a software designer from Company A commented:

I liked that the exercises were short and you were able to mark them as done. You didn’t have to remember where you were going. When you logged in it took you where you were going. So in that respect it was good (app) in my opinion.

However, sometimes the structure caused some confusion. For example, it was difficult to find the same exercise again or to understand where they were in the app. The short exercises were seen positively but there were varying opinions about the introduction videos. For example, the credibility of the videos was questioned and it was difficult to find time to watch them. Performing the exercises at one’s own pace was seen both as a possibility and as a risk. Because participants had to use the app on their own time, performing exercises occasionally at work gave them bad conscience.

### Effects Evaluation

Stress and work engagement (UWES-9) were the primary outcome measures in the effects evaluation. The changes between well-being measures were calculated for the participants who provided information for all the measures both in the beginning and in the end (Table 4). The table demonstrates that the baseline level of the participants was quite good. Stress was significantly lower in Company B in the beginning (Mann-Whitney *U*=40.5, *P*=.03) and TyHy (work community well-being) was significantly higher in Company A both in the beginning (Mann-Whitney *U*=35.0, *P*=.02) and in the end (Mann-Whitney *U*=38.5, *P*=.03). The reliabilities of the measures were calculated using Cronbach’s alpha with the data from the preliminary and final questionnaires. For UWES-9 it was between .94 and .95, .95 for P-TyHy, .96 for TyHy, between .88 and .92 for SWLS, between .89 and .94 for FMI, and between .95 and .96 for WAAQ. For the single-item Stress scale, Cronbach’s alpha could be calculated but showed satisfactory content, criterion, and construct validity for group level analysis [28].

Intervention had no significant effects on well-being in the two companies, neither separately nor when considered as one population calculated by the Wilcoxon test.

**Table 4:** Median effects on the well-being-related measures.

		Company A (n=12)		Company B (n=13)		Both companies (n=25)	
		Beginning	End	Beginning	End	Beginning	End
Stress							
	MED	3.0	3.0	2.0	3.0	3.0	3.0
	IQR	1.8	1.8	1.0	0.5	1.0	0.5
UWES-9							
	MED	4.4	4.3	4.2	4.3	4.2	4.3
	IQR	2.4	2.3	2.1	1.6	2.2	1.8
P-TyHy							
	MED	51.5	51.0	53.3	52.5	51.7	51.3
	IQR	17.9	17.5	12.9	9.4	14.0	10.0
TyHy							
	MED	55.3	56.1	40.6	45.0	47.8	46.7
	IQR	16.8	16.5	12.5	8.6	18.9	19.2
SWLS							
	MED	24.0	24.0	21.0	21.0	24.0	23.0
	IQR	8.5	7.8	8.0	10.0	10.5	7.0
FMI							
	MED	34.0	30.0	35.0	39.0	35.0	36.0
	IQR	12.5	23.5	4.5	6.5	9.0	14.0
WAAQ							
	MED	26.5	23.0	31.0	31.0	29.0	29.0
	IQR	16.0	12.3	8.0	6.0	8.5	11.0

## Discussion

### Principal Findings

This study evaluated the adoption process and effects of a mental health app targeted to individual employees at organizations. Because the findings from a prior pilot study were positive [14], it was expected that the app would be well received and it would have a positive impact on employees’ well-being. However, the adoption rate of the app was low as only a small number of employees (13.0%, 43/332) chose to take part in the intervention, and therefore, the effects of the intervention on the employees’ well-being could not be verified. However, the multiple item questionnaires were tested with Cronbach’s alpha and were proven reliable (alpha >.8).

The assessment of motivations and user experiences shows that employees participated in the intervention and used the app for the "right reasons" (eg, personally motivating reasons). However, the lack of time, the lack of perceived benefits, and the lack of perceived need prevented them from actively using the app and practicing its exercises to improve their stress management skills. Although the app was not targeted only for people under stress or with a diagnosed illness, it apparently failed to bring enough value for mostly healthy people. The app should be developed further in order to concretize its benefits as a preventive approach with immediate positive impact on well-being.

Based on our study, some specific factors that could have a positive influence on the adoption process were identified. Because time was not allocated for employees to use the app at work, its use did not become a part of organizations’ everyday practices. Thus, it would be beneficial to allocate time for app use at work. The HR managers stated that management has to make a decision in principle to adopt the app and only then they can suggest that all employees use the app at work. This finding is in line with earlier studies that have shown that managers’ attitudes influence the intervention success [37].

Management might have more positive attitudes toward the app if it would have proven benefits related to organization’s functions and overall performance of the employees, such as a possibility to measure, monitor, and manage workload or employee welfare. However, the HR managers pointed out that an action plan is needed to describe what to do in risk situations, such as if the employee welfare suddenly decreases. It is also important that the app has proven benefits for the employees and they are communicated properly.

In this study the intervention was delivered through a mobile and a Web app, but the mobile app was not available for Windows phones, which hindered the adoption. It would be important to provide the tools for using the app or develop an app for all the platforms that are used in the end-user organisation. Earlier studies have reported similar findings [38].

Overall, co-operation with HR managers was found to be useful in this study. They were a good source of information for the researchers. It would have been useful to co-operate with them in the concept design phase in order to get a more thorough understanding of the organization’s needs. Moreover, it should be considered to include top management in the intervention planning since they make the strategic decisions. Then it might be possible to make necessary changes in the organisation for the intervention or design them as part of the intervention

Before and during the intervention the employees should have been motivated to participate in the study and use the app, and arranging concrete activities or events at the workplace could have increased the reach and engagement. Earlier research suggests that lack of support outside training sessions can be a barrier for individual or group training in the workplace [38]. Moreover, it is important to avoid burdening employees with data collection activities and rather aim to use the data that is already being collected*.* In this study, it proved difficult to integrate the data collection activities with the existing ones, especially as the researchers were working outside the organization. With an open setting used in this study, it was challenging to engage employees in research procedures, especially final interviews.

There are many possibilities for future research. For example, traditional training methods or well-being campaigns could be combined with digital interventions. Offering physical contact with other people as one intervention component could make also the digital component more engaging [13,39]. In addition, other solutions on how to raise the participation among employees should be studied. Building incentives into wellness programs has been suggested as one solution [5].

### Limitations

This study has some limitations. The small number of participants makes it difficult to assess the effects of the intervention on their well-being and on the organizational settings. Also, the appropriateness of the well-being measures can be questioned, because their sensitivity in the relatively short duration of the study may be suboptimal. Additionally, the open study setting and the uncontrolled participant selection increases the probability of biased results [39]. Even though ACT-based exercises are suitable for everyone for preventing mental health problems and stress, it is not known whether the results would have been different if the intervention would have been targeted for people under stress or with a diagnosed illness.

### Conclusions

This article presents findings from a 4-month study of an individual-level digital mental health intervention at two organizations. Process and effects evaluation from the organization’s point of view was conducted together with the study of employees’ motivations and user experiences. The low number of participants and low intervention adoption show that both the intervention process as well as the digital app should be developed further in order to be successful in this context. Importantly, the study suggests that the intervention planning process should aim to involve the top management of the organization. Although the intervention was not successful as such, the process evaluation provides important insights into how digital interventions should be planned and conducted in the context of occupational well-being. At the organizational level, top management needs to be involved in the intervention planning for fitting into the organization policies, the existing technology infrastructure, and targeting the organizational goals. At the individual level, it has to be tackled how to create time for use and concretise the benefits of the preventive intervention.

## Abbreviations

ACT: Acceptance and Commitment Therapy
FMI: mindfulness questionnaire
HR: human resource
ICT: information and communication technology
P-TyHy: personal well-being questionnaire
SRQ: Self-Regulation Questionnaire
SWLS: Satisfaction with Life Scale
TyHy: work community well-being questionnaire
UWES: Utrecht Work Engagement Scale
VTT: Technical Research Centre of Finland Ltd
WAAQ: Work-Related Acceptance and Action Questionnaire
